# Editorial Comment to Nutcracker syndrome as the main cause of left renal vein thrombus and pulmonary thromboembolism

**DOI:** 10.1002/iju5.12381

**Published:** 2021-09-20

**Authors:** Takanori Sekito, Takuya Sadahira

**Affiliations:** ^1^ Department of Urology Dentistry and Pharmaceutical Sciences Okayama University Graduate School of Medicine Okayama Japan

The current article reported a rare case of left renal vein thrombus (LRVT) and pulmonary thromboembolism (PTE) that were considered to be caused by nutcracker syndrome (NS).^1^ There have been a few case reports of LRVT caused by NS. This case has potential importance to alert urologists to keep in mind that NS can cause thromboembolism, such as LRVT and PTE, which can be fatal.

In this case, the patient’s sedentary job as a long‐distance truck driver was not only a predisposing prothrombotic factor for LRVT, but also a risk factor to exacerbate his flank pain. Sitting for long periods of time can lead to blood congestion causing hypercoagulability and eventually venous thrombosis. Entrapment in the left renal vein (LRV) is an anatomic condition which causes obstruction in blood flow, quite possibly facilitating LRVT. Moreover, compression of the LRV can cause symptoms to manifest as flank pain or hematuria and establish the clinical diagnosis of NS.^2^ The flank pain is visceral pain related to LRV dilation and increased pressure in the LRV. This pain can be aggravated by sitting.^3^

The patient had a severe complication of PTE related to NS in which conservative treatment with an anticoagulant was very effective in resolving the LRVT and PTE. Anticoagulant therapy is indeed the primary approach to treating thromboembolism in patients with NS; however, considering the treatments for NS is also essential for preventing/reducing the recurrence risk of thromboembolism. In patients with NS, depending on the severity of symptoms, individualized treatments are available, such as conservative management, interventional endovascular approaches, and surgery.^4^ With respect to interventional endovascular approaches, placement of endovascular stents is less invasive; however, some complications, such as restenosis, stent migration, and thrombosis, should be noted. A recent paper reported a new and effective method using 3D‐printed extravascular stenting of the LRV for patients with NS.^5^ This novel method is an accurate and personalized treatment for treating NS; however, the optimal treatments for NS have not been established, and additional studies describing long‐term posttreatment outcomes are needed.

## Conflict of interest

The authors declare no conflict of interest.
